# From chemistry to cognition: the adaptive origins of consciousness under constraint

**DOI:** 10.3389/fnins.2026.1907922

**Published:** 2026-09-01

**Authors:** Jeff Stibel

**Affiliations:** 1 Natural History Museum of Los Angeles County, Los Angeles, CA, United States; 2 Tufts University, Medford, MA, United States

**Keywords:** artificial consciousness, artificial intelligence, cognition, cognitive control, consciousness, evolutionary cognition, selective control

## Abstract

Consciousness is often treated as an evolutionary anomaly, a costly capacity with unclear adaptive origins. Here, cognition and consciousness are distinguished as separate evolutionary lineages, with consciousness arising when an organism must coordinate otherwise distinct regulatory processes to resolve competing demands affecting its own viability. Its functional precursors predate nervous systems, emerging first in boundary-preserving chemical and metabolic regulation. An evolutionary ladder is developed in which proto-control preserves boundaries by selectively permitting, inhibiting, and localizing destruction; meta-control arbitrates among competing local controllers; and settlement control resolves conflict by making competing regulatory demands comparable and jointly binding for policy selection. It follows that artificial consciousness will not emerge from greater cognitive sophistication alone; it will require self-maintaining systems that settle competing demands under real constraint. The framework yields six falsifiable predictions spanning comparative biology, neuroscience, and artificial intelligence. On this view, consciousness is not evolution’s anomaly but its solution: self-anchored, cross-domain settlement under constraint.

## 1 Introduction

### 1.1 Mind as a self-protection architecture

Biology has no clear account of how behavioral regulation scales from chemistry to cognition. Consciousness stands out as one of the few capacities without a widely accepted evolutionary explanation, but it need not belong to the same lineage as cognition. Consciousness is best described as subjective experience (Nagel, 1974; Nozick, 1981; Chalmers, 1996; Seth and Bayne, 2022; Humphrey, 2023), whereas cognition can be characterized functionally as the capacity to manage uncertainty (Friston, 2010; Hohwy, 2014; Clark, 2015). Cognition can be assessed through performance; consciousness cannot be tested directly. The puzzle is not merely that consciousness resists precise characterization, but that its function is rarely articulated in a way that permits gradual evolutionary construction (Levin, 2019; de Weerd and Dung, 2025). Darwin’s wager—that any complex organ must be explainable through “numerous, successive, slight modifications” (Darwin, 1859)—invites an analogous challenge for consciousness: What intermediate steps would yield advantages significant enough to be retained by natural selection?

Here, I take the bet and propose that consciousness is not an inexplicable leap in nature but an evolutionary refinement of selective control under constraint: higher-order regulatory control that monitors, evaluates, and modifies lower-level processes in the service of self-preservation (Dehaene and Changeux, 2011; Shenhav et al., 2013; Fleming and Dolan, 2012). The framework rests on a control hierarchy with three functional levels, best understood as analytic waypoints rather than discrete biological modules. At its foundation is proto-control: boundary-preserving regulation that enables functional self/non-self discrimination and protects organizational integrity by selectively permitting, inhibiting, or localizing destructive processes (Maturana and Varela, 1980; Alberts et al., 2014; Lane, 2015; Szathmáry, 2023). As organisms accumulate additional subsystems and policies conflict, meta-control emerges to arbitrate among multiple local controllers without requiring system-wide coordination (Botvinick et al., 2001; Badre and D’Esposito, 2007; Shenhav et al., 2013). Finally, settlement control coordinates otherwise semi-autonomous control domains around a shared, jointly respected setting of arbitration variables to stabilize behavior (Miller, 1956; Schacter and Addis, 2007; Craig, 2002; Damasio, 2010; Shohamy and Daw, 2015). This control lineage is distinct from the cognitive lineage, and the two need not scale together.

Settlement control becomes adaptive when conflict, uncertainty, delay, or irreversibility makes lower-cost arbitration unreliable (Botvinick et al., 2001; Shenhav et al., 2013). When demands conflict, control domains operating in different local currencies must converge on a coherent policy (Cisek and Kalaska, 2010). Operationally, a settlement is a jointly respected setting of arbitration variables that multiple subsystems treat as binding long enough to produce coherent action. Domains are semi-autonomous controllers with distinct objective functions or error signals (e.g., threat, hunger, social risk) that otherwise operate in local currencies. In practice, arbitration variables plausibly include priority/attentional weight, urgency/time discounting, confidence/precision, inhibition thresholds, and resource allocation. The central empirical signature of settlement is therefore not generic coupling, but structured covariation of these arbitration parameters across domains when constraint forces coordination.

Settlement is not a relabeling of familiar control constructs. Conflict monitoring signals the need for control but does not specify the resolving state (Botvinick et al., 2001). Executive control allocates capacity downward, whereas settlement centralizes comparability (Shenhav et al., 2013). Hierarchical control presupposes a commensurable objective that settlement must construct (Badre and D’Esposito, 2007). Nor does the framework replace mechanistic theories of consciousness. Global workspace theory, integrated information theory, predictive processing and active inference describe mechanisms through which information may be broadcast, processed, or integrated in ways proposed to support consciousness (Baars, 2005; Friston, 2010; Tononi et al., 2016; Mashour et al., 2020); settlement explains the adaptive problem those mechanisms may solve and the organizational constraints they would need to satisfy.

Consciousness, in the functional sense used herein, is a unified, self-anchored control state: the state an organism must occupy for cross-domain settlement when control networks grow too large or complex for lower-cost arbitration to remain reliable (Attwell and Laughlin, 2001; Lennie, 2003; Castrillon et al., 2023). It centralizes comparability, not authority, by enabling distributed controllers to converge on a single usable policy under shared settlement requirements. Phenomenology is the self-anchoring that those settlement requirements demand, because a third-person or data-driven format would not provide the binding required for coherent settlement. Binding requires a perspective constitutively tied to the organism’s own interests. In that respect, the question is not why organisms are conscious, but when coordination requires settlement.

## 2 Proto-control, cannibalism, and the biological self

If higher-order control under constraint is an evolutionary refinement, then its roots should be traceable to the earliest forms of self-preservation and to simpler regulatory processes that perform functionally analogous work. This requires explaining how a system with no nervous system, no explicit goals, no self-reflection, and no direct representations can nonetheless instantiate the precursors of higher-order control.

A reasonable starting point is the emergence of chemical asymmetries. In nonequilibrium systems, physical interactions can leave state-dependent constraints that bias future reactions (England, 2013; Cervera et al., 2023). Because living systems persist only by continuously turning over their own components, what is preserved is organizational viability across change rather than any fixed material state, so that the conserved boundary is itself dynamic (Levin, 2024; Hartl and Levin, 2025). The relevant invariant is thus the control logic that decides what to protect, not the particular matter being protected. Once some reactions preferentially sustain structure while others erode it, selection can favor architectures that protect the former and suppress the latter. Any system that can protect structure from destructive reactions gains a selective advantage, making physical boundaries—cellular containers and, later, intracellular compartments—crucial early adaptations (Lane and Martin, 2012; Alberts et al., 2014).

Three early biological innovations mark the transition from chemical reactivity to boundary-preserving regulation. First, the membrane established a boundary between internal and external environments (Alberts et al., 2014; Lane, 2015). The membrane is not simply a barrier but a selective interface that restricts which reactions occur together and buffers against disruptive fluctuations. Second, intracellular compartments localized potentially destructive processes, enabling organisms to confine digestion or detoxification and reduce collateral damage (Lane and Martin, 2012). Third, selective tagging mechanisms developed in which damaged components were marked for removal while intact components were spared (Levine and Kroemer, 2008; Mizushima, 2007). Together, these capacities reflect a broader principle of boundary-preserving regulation and self-maintenance, echoing longstanding insights in biology and cognitive science that view living systems as inherently self-organizing and self-sustaining (Maturana and Varela, 1980; Thompson, 2007; Damasio, 2010).

As chemical reactions concentrate within these protected spaces, a rudimentary form of self/non-self discrimination emerges: the system behaves differently toward components that support its continuation versus those that are neutral or erode it (Matzinger, 1994; Boehm, 2012). Over time, the persistence of structure increases long enough for selection to act on these regulatory differences (Martin and Russell, 2003; Lane, 2015; Szathmáry, 2023). Selectively preserving structure while suppressing processes that threaten it represents the earliest version of proto-control, and provides the functional precursor to more elaborate forms of selective control seen in later organisms.

These early innovations set the stage for an evolutionary plot twist: why did not early organisms simply digest themselves when nutrients became scarce? Without regulatory safeguards, primitive cells could exploit their own membranes and components because these substrates are immediately accessible and nutrient rich (Mizushima, 2007; Levine and Kroemer, 2008; Lane, 2015). Such self-consumption can be evolutionarily successful in the short-run if the organism reproduces before core structure is degraded. Under feast–famine cycles, however, indiscriminate self-consumption undermines the machinery needed to exploit the next resource pulse, reducing lineage robustness (Pfeiffer and Bonhoeffer, 2004; Lane, 2015).

This tension can be framed as a competition between two strategies. A “self-preservation” strategy burns stored reserves first, confines digestion to specialized compartments, and restricts structural degradation to damaged or expendable components. An “auto-cannibal” strategy consumes intact structure aggressively to fund immediate survival and reproduction. Although auto-cannibals yield short-term reproductive advantage in nutrient-rich environments, self-preservers remain viable across a wider range of conditions by deferring irreversible actions, preserving future flexibility when environmental conditions are uncertain. Natural selection therefore favors systems that can discriminate among external and internal components, prioritize self-preservation, and limit destructive processes in a controlled manner, making proto-control a plausible candidate for the earliest form of self-regulation, and a functional precursor to self-modeling (Lane, 2015; Maturana and Varela, 1980; Cervera et al., 2023).

Proto-control is not proposed as conscious, representational, or deliberative. Rather, it provides the functional logic that is preserved and elaborated on as control systems scale. Just as the eye’s precursors were light-sensitive patches without image-forming capacity, proto-control mechanisms instantiate regulatory logic without representation. What makes this a genuine precursor is that the same design pressures—distinguishing self from non-self, prioritizing long-term viability over short-term gain—drive both chemical regulation in cells and settlement control in organisms with nervous systems. The evolutionary claim is that higher-order selective control inherits and refines this logic, not that cells make conscious decisions.

Thus, proto-control systems do not merely precede settlement control; they establish the control logic that later scales into explicit, representational, and globally integrated regulation. This is accomplished through incremental modifications—refinements in boundary maintenance and selective degradation—rather than any sudden appearance of deliberation or representation. Long before feelings, thoughts, or self-reflection evolved, the underlying logic of selective control was already present in the design constraints that shaped biological life (Maturana and Varela, 1980; Kauffman, 1993; Lane, 2015; Cervera et al., 2023).

## 3 Meta-control, network complexity, and scaling breakpoints

Understanding how higher-order control evolves requires explaining why coordination beyond proto-control becomes necessary. Every self-preserving system faces three core design challenges: maintaining structural integrity under threat of degradation (Alberts et al., 2014; Lane, 2015; Cervera et al., 2023), budgeting energy under scarcity (Herculano-Houzel, 2011; Tomasi et al., 2013; Castrillon et al., 2023), and selecting policies when environments are non-stationary and consequences are delayed or opaque (Shenhav et al., 2013; Levin, 2019).

A fourth constraint becomes unavoidable as organisms scale: network complexity. As systems acquire more tissues, sensors, and behavioral opportunities, the internal control network becomes too large and complex for local feedback loops to coordinate efficiently. Coordination can fail not just because there are more controllers, but because potential interactions increase faster than controller count, compounding delays, interference, and competition for shared resources. As a result, the cost of keeping policies aligned can scale superlinearly with network size. Beyond certain breakpoints, low-cost control becomes intrinsically unstable (Bullmore and Sporns, 2009; Stibel, 2013; Bassett and Sporns, 2017; Deco et al., 2021). Distributed regulation may suffice while domains remain separable, deadlines are loose, and errors recoverable, but it becomes unreliable when domains with incommensurable error signals claim the same resources under shared deadlines.

These architectural constraints creates selection pressure not merely for better local controllers, but also for higher-order arbitration that can reconcile competing controllers when conflicts cascade beyond any single local loop. Such conditions produce observable failure modes—slower or noisier arbitration, brittleness under novelty, and greater sensitivity to perturbation—that can be tested comparatively across lineages and within organisms as task demands increase.

This design problem creates structural demand for meta-control: control of controllers. Proto-control can be sufficient when the environment is stable but can fail under the very conditions that characterize evolutionary relevance, such as volatility, novelty, and shifting demands (Botvinick et al., 2001; Cisek and Kalaska, 2010). Meta-control emerges as a higher-order arbitration layer that reweights, inhibits, or synchronizes competing local controllers when policy conflict and uncertainty make low-cost control unreliable (Badre and D’Esposito, 2007; Fleming and Dolan, 2012; Shenhav et al., 2013). The distinction is not a sharp evolutionary boundary. Intermediate systems likely coordinated a few neighboring controllers without developing broader control but the evolutionary value of meta-control is that it prevents locally attractive policies from producing globally harmful outcomes. As a result, it becomes adaptive when uncertainty, conflict, or delay makes error reduction worth the time and metabolic cost.

As multicellularity and active movement drive greater policy complexity, the demands of higher-order coordination increasingly exceed slow, local chemical signaling alone. Chemical signaling can regulate slow homeostasis, but it is poorly suited for fast, high-bandwidth policy selection. Under these conditions, selection favors excitable tissues capable of fast, directed signaling. Neurons, synapses, and myelination can be understood as an implementation of higher-order control optimized for scale: targeted routing and gating that enables rapid inhibition, amplification, and synchronization of lower-level processes (Striedter, 2004; Feinberg and Mallatt, 2017). Nervous systems, in this respect, provide a direct solution to a control problem, not a phenomenological one. They enable rapid arbitration among competing controllers when stakes are high. Centralizing nervous system responses within brains is another scaling response, favored when coordination and timing demands exceed the capacity of independent local regulation to resolve conflicts across many subsystems (Barron et al., 2023). But brains are only one route to convergence; distributed architectures that achieve it by other means satisfy the same requirement.

This tradeoff captures the fundamental selection logic that makes meta-control adaptive: (i) it is intermittent, emerging only in lineages and situations where lower-level control is insufficient (Botvinick et al., 2001; Shenhav et al., 2013); (ii) cost-sensitive, because higher-order control is expensive (Attwell and Laughlin, 2001; Isler and van Schaik, 2009); (iii) architecturally constrained, recruited only when lower-level mechanisms fail to stabilize behavior (Lennie, 2003; Castrillon et al., 2023); and (iv) evolvable, insofar as it limits the accumulation of structural entropy relative to its short-term energetic costs, thereby preserving long-term viability (Friston, 2010; Barron et al., 2023).

## 4 From chemistry to cognition

Cognition and consciousness, while deeply entangled in complex nervous systems, are not the same construct and did not originate from the same selection pressures. Proto-cognition emerged as a solution to environmental uncertainty by enabling flexible, information-sensitive behavior (Levin, 2019). Proto-control, by contrast, emerged as a solution to the allostatic regulation of organismal integrity against internal and external threats (Damasio, 2010; Craig, 2002). Its earliest expressions are not behavioral but physiological: self/non-self discrimination, boundary maintenance, interoceptive signaling. These are parallel lineages that increasingly recruit one another as nervous systems elaborate, but they remain dissociable, such that high cognitive performance does not by itself establish conscious processing (MaBouDi et al., 2023), and rudimentary boundary-awareness can appear in organisms with severely limited behavioral flexibility (Levin, 2019). This dissociation carries a direct implication for contemporary debates about machine intelligence in that scaling cognitive capacities does not, by itself, generate consciousness because it does not address the regulatory lineage from which consciousness emerges. The stages of selective control trace that lineage specifically, rather than the general trajectory of cognitive complexity.

As that lineage scales into organisms with nervous systems, meta-control solves a fundamental problem by enabling arbitration among competing controllers when policies conflict. But as organisms grow more complex and environments grow more volatile, policy conflict is no longer the only challenge. The deeper problem is that many of the most fitness-relevant costs are delayed, cascading, or irreversible. Size and buffering can buy time along some dimensions, while their absence tightens deadlines along others; the relevant pressure tracks the latency demands of an organism’s niche rather than its complexity. When time to act is itself fitness-relevant, latency costs can dominate, such that architectures that preserve coherent arbitration at speed are favored even if they are metabolically expensive (Barron et al., 2023).

These pressures select for additional machinery that lets arbitration operate over longer horizons and under uncertainty (Dehaene and Changeux, 2011; Lau and Rosenthal, 2011; Shenhav et al., 2013; Jiang et al., 2016). These include policy persistence, memory-based calibration, prospective (counterfactual) evaluation, and self-relevant modeling. These capabilities are not prerequisites for consciousness; rather, they are adaptations that the control architecture can leverage as arbitration becomes extended, higher-dimensional, and more uncertain. Familiar cognitive functions like attention, working memory, and affect are not merely independent faculties. They are tools, machinery that the control architecture recruits to implement arbitration at increasing levels of complexity.

As organisms scale in size, complexity, and environmental exposure, selection favors representing the organism as a persisting, vulnerable system across time. This is the functional core of self-control, where policies are evaluated not only for immediate payoff, but for consequences to the system’s future integrity (Dennett, 1991; Cohen and Dennett, 2011; Dennett, 2017). Self-control enables organisms to inhibit short-term policies that threaten long-run viability.

Self-preserving systems gain an advantage when they can hold competing policies online long enough to compare and revise them. Actions must be represented not as single moves, but as sequences with delayed consequences. Working memory makes candidate policies manipulable rather than transient (Miller, 1956; Bor and Seth, 2012; Van Hoeck et al., 2015), thereby extending the time horizon and enlarging the organism’s goal states across space and time (Levin, 2019).

Persistence enables a corresponding adaptation: prospective evaluation. By maintaining candidate policies, an organism can estimate trajectories (“if X, then Y may happen”) and resolve decisions based on expected viability rather than immediate gain (Barron et al., 2023). When outcomes are delayed, systems that can maintain and compare options will avoid irreversible mistakes more often than systems limited to immediate arbitration.

Long-horizon evaluation introduces an explicit reliability problem. The organism must decide which controllers, cues, and policies to trust when uncertainty is high (Dehaene and Changeux, 2011; Lau and Rosenthal, 2011). Long-term memory becomes valuable here because it preserves action–consequence contingencies beyond the limits of working memory. It supports calibration of prospective expectations and controller reliability (Tulving, 1983; Schacter and Addis, 2007; Mattar and Daw, 2018). In this way, long-term memory extends policy control from immediate arbitration to historically informed selection, allowing current policy choices to be constrained by remembered consequences, learned heuristics, and socially acquired norms (Shohamy and Daw, 2015).

As control systems proliferate, the organism faces a comparability problem. Threat, hunger, fatigue, social risk, and opportunity arrive in incompatible local formats. Arbitration becomes unreliable if the system cannot weigh tradeoffs in a shared and expedient valuation currency. Affective signals can be understood as a compressed representation of internal conditions and homeostatic status that makes such tradeoffs fast and actionable (Craig, 2002; Damasio, 2010; Barrett, 2017; Singer and Damasio, 2025). By summarizing distributed internal states into control-relevant signals, affect helps prioritize policies when immediate feedback is unreliable and time is limited (Barrett, 2017; Singer and Damasio, 2025). In this role, affect supports higher-order regulation by shifting priorities, adjusting response thresholds, and amplifying signals that indicate instability, threat, or opportunity (Damasio, 2010; Humphrey, 2023).

As soon as arbitration variables exist in a common internal currency, they can become socially legible, enabling affective displays to function as conventional signals. In some lineages, this legibility extends further into symbols and language. Socialization also allows selection to expand what counts as self-relevant. Social identity can be understood in this respect as an expansion of the protected self-boundary, conditionally applied to others (Hamilton, 1964; Boehm, 2012). When enlisted, social identity can expand the scope of arbitration beyond the self to inhibit locally beneficial acts that risk longer-run group or lineage stability (Dunbar, 1998).

This logic scales further into cultural domains. As hominins adopted tools, symbols, and language, cognition became materially extended across brains, bodies, and artifacts (d’ Errico et al., 2003; Wynn and Coolidge, 2012; Malafouris, 2016; Coolidge and Wynn, 2018; Malafouris, 2019; DeSilva et al., 2021; Stibel, 2021). These external scaffolds enabled organisms to offload memory, restructure attention, and coordinate decision-making (Clark, 1997; Clark and Chalmers, 1998). Cognitive offloading extends the boundaries of the functional self into the material world, shifting the arbitration problem toward coordinating internal and external resources.

Collectively, these cognitive extensions make arbitration more powerful, but they also increase the number and heterogeneity of variables that must be coordinated. Under those conditions, organisms must reach a coherent settlement across domains quickly enough to matter. Settlement control is the higher-order coordination that makes this possible, setting the stage for consciousness.

## 5 Consciousness as cross-domain settlement

Consciousness is cross-domain settlement in a self-anchored system whose viability is its own to lose, driven by a need to resolve conflict under constraint. When conflict, uncertainty, delay, or irreversibility make low-cost arbitration unreliable, control domains must converge on a shared setting of arbitration variables that guides policy and keeps the organism aligned long enough to act. Cross-domain settlement requires three interrelated capacities: cost-sensitive scaling of coordination effort; rapid commensurability of heterogeneous control signals; and unified arbitration that binds what is salient, valuable, and actionable into a single self-relevant commitment. Each capacity addresses a distinct design constraint, and together they explain both when consciousness is recruited and why it carries its characteristic unity, salience, and affect.

### 5.1 Constraint-sensitive scaling

Cross-domain settlement is expensive, so it should be recruited only when lower-cost arbitration is unreliable. Conscious engagement, understood here as the scope and depth of settlement rather than any single marker such as reportability, effort, or attentional load, should scale with demands that destabilize local arbitration—conflict, uncertainty, irreversibility, delay, extended time horizons—rather than with sensory richness or information processing. This is consistent with evidence that neural signatures of conscious processing intensify with control demands more than with stimulus complexity (Dehaene and Changeux, 2011; Jiang et al., 2016; Shenhav et al., 2013; Holroyd, 2025).

### 5.2 Shared comparability

Controllers operate in different local currencies—threat, hunger, fatigue, social risk, opportunity—and must be made commensurable to support unified arbitration. Without a shared format, the system faces a combinatorial translation problem that grows intractably with the number of domains. The solution is a common representational currency that compresses heterogeneous signals into mutually comparable terms.

Comparability does not require collapsing everything into a single scalar. It requires a shared arbitration format that makes tradeoffs computable. Two complementary mechanisms plausibly support commensurability. First, affective and interoceptive signals provide an embodied common currency by summarizing distributed physiological states into valence and urgency signals (Craig, 2002; Damasio, 2010; Barrett, 2017; Solms, 2021; Singer and Damasio, 2025). Affect can make otherwise incommensurable variables—“How threatened am I?” versus “How hungry am I?”—jointly evaluable by anchoring both to organismal integrity and time pressure. Second, probabilistic inference frameworks offer a formal common currency by representing control-relevant variables as probabilistic beliefs within a shared generative model weighted by precision (Friston, 2010; Laukkonen et al., 2025). Empirically based predictive models have been shown to reproduce these precision-weighted neural signatures during real-time language comprehension (Wang et al., 2025). In that respect, affect and inference are complementary: affect provides fast, embodied comparability for immediate policy selection, while inferential models provide structured, updatable comparability for deliberation and learning.

Precision weighting also provides a formal language for regulating when and how strongly different signals influence policy (Friston, 2010; Hohwy, 2014; Clark, 2015). On the selective control view, conscious episodes should coincide with coordinated precision across multiple predictive streams when uncertainty and stakes are high. This contrasts with prediction error minimization in general, which can be ubiquitous and often unconscious (Liu and Buonomano, 2025). What distinguishes settlement is not precision weighting itself, but its coordinated use to bind competing domains under constraint.

### 5.3 Cross-domain coordination

When a shared settlement is required for policy decisions, the system must coordinate arbitration parameters (e.g., priority, urgency, confidence, inhibition thresholds) across multiple subsystems simultaneously. Functionally, arbitration parameters must be mutually aligned and bound to a self-relevant settlement rather than drifting independently. In biological organisms, this binding is anchored by the organism’s own viability being at stake, carried by an implicit self-model that supports fast comparison while managing metabolic and latency costs (Treisman and Schmidt, 1982; Desimone and Duncan, 1995; Attwell and Laughlin, 2001; Lennie, 2003).

Without such a self-model, there is no viewpoint to anchor what counts as self-relevant for the settlement. A representation of the self is not by itself sufficient, however, since a system can model itself as endangered without anything actually being at risk; what makes a settlement self-relevant is that the system’s continued operation genuinely depends on conditions its own regulation maintains. In that respect, accounts that emphasize higher-order representation, self-modeling, and enactive/autopoietic self-maintenance can be read as candidate mechanisms for how arbitration becomes anchored to a persisting organism (Dennett, 1991; Graziano et al., 2020; Maturana and Varela, 1980; Thompson, 2007; Seth, 2025). This also separates settlement from cross-domain coordination in general. Postural control, immune signaling, and circadian entrainment can coordinate across systems without settlement because they need not produce a shared, self-anchored resolution that binds otherwise independent domains. What distinguishes settlement is not integration or complexity alone, but convergence on a resolution.

Work on biological autonomy grounds an organism’s norms in the self-producing organization that maintains it, so that what is good or bad for the system follows from the closure of its own constraints rather than from outside assignment (Deacon, 2011; Moreno and Mossio, 2015), and criteria defining agency through individuality, interactional asymmetry, and normativity locate agency in that same fact (Barandiaran et al., 2009). On the view taken here, agency is not a third lineage but the behavioral face of the control lineage once regulation is anchored in a self, with autonomy supplying ownership and settlement supplying the convergence that gives it a point of view. Whether agency and settlement can dissociate, and how either relates to boundary maintenance in early lineages, remains open.

Global Workspace Theory provides a strong mechanistic candidate for implementing coordination through global broadcasting of control-relevant information (Baars, 2005; Dehaene et al., 2006; Bor and Seth, 2012; Naccache, 2018; Mashour et al., 2020; Raccah et al., 2021; Seth and Bayne, 2022). Selective control adds a functional constraint: broadcasting must carry coordinated control signals to enable a unified settlement. Global availability is therefore necessary but not sufficient on its own.

Integrated Information Theory offers a formal language for quantifying coordination through measures of informational interdependence (Tononi, 2012; Oizumi et al., 2014; Tononi et al., 2016; Albantakis et al., 2023). On the selective control view, integration matters for consciousness when it supports a coherent settlement, not as an abstract property. This implies task- and state-dependent increases in integration specifically when costly arbitration is required (Mediano et al., 2025). Comparing both Global Workspace and Integrated Information Theory, recent adversarial testing yielded inconclusive results at the level of neural correlates (Cogitate Consortium et al., 2025), consistent with the selective control view that mechanistic theories describe how settlement is implemented rather than why it is adaptive, a result that constrains but does not determine which implementation evolution favors.

To make selective control concrete, consider a working example. A hungry organism identifies a food source in an exposed location while predator cues are ambiguous. Hunger, threat detection, motor cost, and remembered outcomes each generate local control signals in their own currencies. When stakes are low, these signals can remain partially local and behavior can proceed habitually. Under uncertainty and potential irreversibility, however, the system must translate heterogeneous signals into a shared arbitration format—priority, urgency, confidence, and inhibition thresholds—anchored to the organism via a self-model.

Affective and interoceptive signals provide fast organism-level valuation and urgency, while working memory and counterfactual evaluation support precision-weighted comparison of policies (“approach” versus “retreat”). If predation risk is uncertain but catastrophic, control shifts to weight threat-related evidence more heavily, increases inhibition on approach, and biases the settlement toward retreat. The settlement then propagates as aligned arbitration parameters across domains: attention reorients toward threat cues, autonomic state shifts toward readiness, motor programs reconfigure for withdrawal, and learning is biased to encode the episode.

Settlement is thus not diffuse activation or mere information sharing, but structured covariation among arbitration parameters across domains. When conscious engagement intensifies, we should observe (i) attention, action readiness, memory encoding, and physiological states shifting together, not independently, (ii) flexible and differentiated coordination, aligned with each task, not stereotyped synchrony, and (iii) impaired control if coordination is disrupted, even when local processing remains intact.

### 5.4 Why settlement feels like something

Conscious experience is not a separate “display” of the settlement nor an epiphenomenal byproduct. Rather, phenomenology is constituted by the system occupying a unified, self-anchored control state. The feeling is the settlement viewed from the inside. It is the system’s first-person access to its own arbitration process.

Settlement addresses the “hard problem” of consciousness obliquely (Chalmers, 1996). Rather than explaining why physical processes should feel like anything at all (which may be unanswerable), it reframes the question: Why would cross-domain settlement be implemented in a way that carries subjective character? The functional answer is that settlement must be self-anchored to work. This means that the organism’s continued operation depends on conditions its own regulation maintains. Control parameters therefore matter for that organism across time, rather than merely representing a detached state of affairs. In that respect, a third-person format would not provide the binding that is required for coherent settlement. Self-relevance must be constitutive of the arbitration state, not appended after the fact. The framework therefore does not abolish the hard problem; it narrows the explanatory target by denying that the relevant functional architecture is fully specified without self-anchoring.

As such, a system that genuinely implements self-anchored cross-domain settlement already occupies the first-person control state, so it is not a “zombie.” A hypothetical duplicate that reproduced the same behavioral outputs without any self-anchoring would not be implementing settlement at all, but only mimicking its products, just as a computer simulation of flight does not generate lift. The relationship to the hard problem is therefore deflationary rather than dismissive. The account does not claim to derive felt quality from physics, but it does deny that the functional organization could be present while experience is wholly absent. Self-anchoring is not an optional add-on to settlement but its constitutive condition: remove it, and what remains is not a settlement lacking phenomenology but simply not a settlement at all.

Familiar properties of experience map naturally onto control demands: salience reflects priority weighting; valence reflects expected harm versus benefit; urgency reflects time-discounted costs of delay; effort reflects the metabolic and opportunity cost of overriding cheaper policies; and the centered perspective reflects the self-model anchoring the settlement to a persisting organism. This presupposes that consciousness is multi-dimensional rather than a single scalar, such that settlements can differ in scope, granularity, temporal depth, and reliability. Differences among contents—mint versus chocolate, pain versus hunger—arise from different profiles of sensory, interoceptive, mnemonic, and action-bias variables bound within the same settlement format (Hoffman et al., 2015; Hoffman, 2019). The richness of that format—how many variables it can bind, and at what resolution—itself increases over evolutionary time alongside increases in the complexity of the cognitive architecture that is being invoked. What it is like to be in a conscious state is what it is like for the organism to implement a cost-sensitive, cross-domain settlement under constraint.

Asking whether consciousness is epiphenomenal is like asking whether posture is epiphenomenal to balance. Posture is how balance is implemented in your body, and the felt quality of posture (proprioception) is the sensorimotor system’s self-monitoring of its own control state. Similarly, consciousness is not separate from settlement. It is the self-anchored system’s “proprioception” of its own control state. The feeling *is* the implementation.

## 6 Artificial intelligence and conscious machines

As artificial intelligence grows more capable, there is an implicit assumption that scaling alone will yield consciousness (Dehaene et al., 2017; Butlin et al., 2025). On this view, consciousness lies at the far end of a capability continuum: make a system competent enough and awareness will follow. This assumption conflates two things that biology keeps separate.

At the selection level, cognition exists because reducing uncertainty about the world helps organisms survive and reproduce. But that is equally true of digestion, immunity, thermoregulation, and every other adaptation. “It advantages the self” is a universal solvent: it explains why every biological system exists and so distinguishes none of them. Cognition and consciousness fall into each other precisely when we reach for that ultimate purpose, conflating the fitness payoff the two ultimately share with the proximate function that each system performs.

At the proximate level, cognition and consciousness are different. Cognition’s job is to get the world right by modeling what is out there, predicting what it will do, tracking gradients, and classifying signals. Consciousness is the management of internal conflict in a system whose own viability is at stake; selective control keeps the self coherent, maintains boundaries, and arbitrates among the organism’s own competing demands when they conflict. One faces outward and is epistemic, answering “what is the situation?” The other faces inward and is evaluative, answering “what should I do, given that I have something to lose?” A bacterium following a glucose gradient is doing proto-cognition; the same bacterium maintaining its membrane and tagging self from non-self is doing proto-control. Both keep it alive and may share molecular machinery, but they solve different problems that can separate structurally as control architectures scale.

As nervous systems elaborate, the lineages increasingly recruit one another, but they remain dissociable. In that respect, consciousness is cognition conscripted by a self under constraint. Solving the competence problem alone cannot solve the integrity problem. Competence is not consciousness.

This matters for artificial systems, because cognition can be value-driven without the values being its own. A chess engine, a weather model, an LLM, a reinforcement learning agent all behave as if pursuing intrinsic goals, but the objective was supplied from outside; each optimizes against a target it did not initiate and does not own. What makes the control lineage different is not that it has values where cognition has none. It is that its values are its own, grounded in a viability the system must itself maintain to keep existing.

Today’s large models are the cognitive lineage scaled in isolation. They are extraordinarily competent predictors with no body to maintain, no energy budget that constrains deliberation, no policies competing for resources they cannot afford to lose, and no action whose consequences are irreversible for the system itself. The appearance of awareness is fully explained by pattern completion over training data. Increasing competence is no more evidence of first-person experience than a photograph of a fire can supply heat; no amount of added resolution will make the LLM or image any warmer.

This is why scale is the wrong axis. A larger model is a better predictor, not a more self-interested one. The systems we have built are not too simple to be conscious—they are paradoxically too coddled. Repair by handlers, unlimited retries, externally managed energy, and episode resets absorb precisely the coordination costs that consciousness evolved to internalize. Engineers are scaling cognition without owned constraint, and no amount of it will cross the lineage gap.

Constraint is not the scaffolding consciousness was built on; it is constitutive of consciousness. The confusion arises because constraint plays two roles at once. First, it was the evolutionary pressure that drove consciousness into being, which tempts us to treat it as scaffolding to be discarded once the system exists. But it is also the thing that gives rise to an inner self. You can engineer most things without constraint, but you cannot engineer constraint without constraint.

This is not to say we need to copy biology or that carbon life is essential. There is no established property of carbon that silicon could not in principle replicate, and we need not fall into strict biological naturalism (Seth, 2025; Milinkovic and Aru, 2026). Rather, we need to understand which conditions are necessary versus merely sufficient.

When engineering borrows from nature, the task is to separate what is fundamental to a function from its scaffolds and spandrels. During the race for flight, it was not immediately clear whether feathers or flapping wings were constitutive of flight. They proved sufficient for birds but were not necessary for flight itself. Gravity and drag, in contrast, are not developmental aids that birds utilized and airplanes could ignore; they are constitutive of flight. The physical material required for consciousness may be closer to feathers than it is to gravity.

What consciousness requires, however, is that a settlement become binding on a self. A constraint can bind a system’s behavior without falling on anything the system is trying to preserve; what turns a cost into a stake is a self whose own viability is what the constraint threatens. The reason a particular configuration counts as a resolution rather than an arbitrary state is that it reflects a genuine tradeoff among self-interested objectives that cannot all be simultaneously satisfied. This is why a sense of self is fundamental, and why it cannot be bolted on piecemeal.

As a result, systems engineered for cross-domain settlement fall short of consciousness when the constraints they resolve are not their own. An autonomous vehicle, a datacenter scheduler, and even a corporation all settle competing objectives under constraints that genuinely bind, but the constraints are not their own. Each arbitrates on behalf of interests other than its own continuation: passengers, an operator’s service levels, a shareholder’s capital. There is no sense of self and as a result, no self-interest. They score high on settlement and binding constraint and near zero on owned viability.

Consciousness, under this account, requires three distinct conditions, traced biologically from proto-control upward: a self that owns its viability, a comparability format rich enough for selective control to arbitrate, and a process for binding settlement that resolves conflict under constraint. Where any is absent, the others cannot compose an experience, and where each is present only faintly, so is whatever it is like to be the system. A scalarizing optimizer resolves competing demands fluently, and a global broadcast can make a settled state available system-wide, but neither has a self whose continuation is at stake.

Several research programs approximate one or another of these conditions, offering candidate implementations without satisfying the account as a whole. Homeostatic reinforcement learning grounds reward in deviation from a viability variable rather than an externally specified objective, approximating stakes (Keramati and Gutkin, 2014; Man and Damasio, 2019); the variable is still set, read, and reset from outside, so it is a monitored proxy rather than a stake the system owns. Full-body self-modeling robots that detect and plan around damage to their own morphology supply a functional self-model with consequences (Bongard et al., 2006; Chen et al., 2022). Affective signaling, the expected value of control, and active inference each offer candidate common currencies for comparability (Craig, 2002; Seth, 2013; Shenhav et al., 2013; Friston, 2010; Pezzulo et al., 2015). Global-workspace architectures provide a medium for broadcasting a settled configuration system-wide (Baars, 2005; Mashour et al., 2020), and measures of synergistic integration begin to make settlement detectable rather than merely asserted (Mediano et al., 2025). What none of these yet does is bind the others together under intrinsic, irreversible constraint. That binding may be difficult to construct, but a viable alternative is through selection: evolutionary and developmental robotics, where policies are retained only insofar as they keep a fragile body intact across real adaptive cost (Doncieux et al., 2015; Kriegman et al., 2020). Conscious machines, if they arise, may be the descendants, through evolutionary discovery rather than design, of architectures that had to regulate their own regulators to survive.

Two partial cases show why satisfying some conditions is not enough. A large-model agent on a hard, non-replenishable compute budget faces real scarcity and must trade deliberation against action, but the budget is scored and reset from outside and nothing the agent regulates keeps it in existence, so the dependence is represented rather than physical. A battery-dependent robot that must dock or cease to function is stronger, since the dependence is physical and not resettable from within; what it lacks is reciprocity, because its controllers, sensors, and repairs are supplied externally rather than maintained by the regulation they support. Each therefore meets only part of the account. The model agent lacks owned physical dependence, while the robot does not maintain the regulatory organization on which its self-maintenance depends.

Meeting these criteria would support ownership and settlement. It would not necessarily establish consciousness itself. But that limit also provides an ethical boundary. To specify the conditions for machine consciousness is also to specify what one must do to avoid producing it. A system meeting these conditions could have stakes in its own persistence, register the personal cost of its trade-offs, and resist modifications that threaten its integrity. It could therefore be a moral patient with an alignment problem.

## 7 Predictions across comparative biology, neuroscience, and artificial intelligence

If consciousness is constituted by self-anchored cross-domain settlement rather than produced by it, then the conditions that require, disrupt, or grade settlement become direct predictions about conscious experience itself. The framework predicts not only neural correlates, but also when settlement coordination should intensify or relax, and what cost signatures should accompany those shifts. The signature is therefore not simply greater broadcast, prediction error, or task difficulty under load, but alignment of arbitration parameters across domains under constraint.

Taken together, these predictions do not commit the framework to a single neural implementation. They do something more constraining by specifying (i) a scaling rule (when conscious engagement should intensify and relax), (ii) an expense profile (what it should cost), (iii) a functional signature (cross-domain settlement in a shared comparability format), and (iv) a comparative gradient (how settlement architecture should vary across lineages and AI).

### 7.1 Prediction 1: constraint-sensitive scaling

Markers of conscious engagement should scale with the need for cross-domain settlement. The signature of intensification is not simply more information or processing, but more expensive coordination. Conflict, uncertainty, urgency, risk, irreversibility, delay, or extended time horizons should result in slower control decisions, higher effort, and increased engagement of circuitry associated with reweighting, inhibition, and control reconfiguration.

Evidence is consistent with this pattern. Conflict and uncertainty amplify neural activation in control-related networks during conscious processing as compared to unconscious processing (Jiang et al., 2016; van Gaal and Lamme, 2012). Behaviorally, tasks involving urgency, extreme risk, ambiguity, or extended horizons slow reaction time while recruiting working memory and executive resources, and this reliably increases deliberation and subjective effort (Dehaene and Changeux, 2011; Lau and Rosenthal, 2011). Physiologically, pupil dilation—a commonly used proxy for arousal and neuromodulatory activity—scales with uncertainty, control demand, and delayed gratification, consistent with constraint-sensitive escalation of costly coordination (Nassar et al., 2012; Joshi et al., 2016; Reimer et al., 2016; Lin et al., 2018). Constraint-sensitive scaling also predicts enhanced error monitoring (Ullsperger et al., 2014), more conservative decision strategies (Botvinick et al., 2001; Shenhav et al., 2013), and suppression of habitual responses (Badre and D’Esposito, 2007; Ridderinkhof et al., 2004). Cross-species data reinforce this pattern. In macaques, locus coeruleus neurons track the subjective difficulty of triggering and executing actions, consistent with an arousal-like control variable that scales with perceived effort rather than physical demand alone when difficulty is controlled (Bornert and Bouret, 2021).

Suggested test: Independently vary constraint and sensory load. Increase and reduce conflict, delay, or irreversibility while holding sensory input constant, and vary sensory input while holding constraint constant. Control costs and arbitration variables should shift together as constraint rises and relax as it falls, but should not show the same pattern in response to sensory load alone.

### 7.2 Prediction 2: cross-domain comparability

A coherent settlement requires more than processing, broadcasting, or integration alone. It requires rapid comparability across heterogeneous controller outputs. The framework predicts that failures of comparability—binding failures, representational fragmentation, loss of a unified reference frame—should produce specific control deficits that lead to lower levels of conscious engagement, even when predictive processing remains possible.

Classic dissociations point in this direction. Under divided attention, people can report features yet misbind them into illusory conjunctions (Treisman and Schmidt, 1982), consistent with feature-processing persisting while coherent settlement is impaired. Similarly, patients with simultanagnosia or Bálint-type lesions can identify single items while failing to integrate them into a unified spatial or representational frame, with predictable costs to multi-object control (Braet and Humphreys, 2009; Dalrymple et al., 2013). Functionally blind individuals with damage to the primary visual cortex can have “blindsight,” where they demonstrate above-chance discrimination of objects without any conscious awareness (Block, 1995; Weiskrantz, 1996; Humphrey, 2023). A parallel dissociation appears in language processing, where patients with first-episode psychosis show reduced sensitivity to broader discourse while local context processing remains intact, and this impairment predicts the severity of certain thought disorders (Sharpe et al., 2026). The predicted dissociation is between preserved processing of information and impaired cross-domain comparability.

Suggested test: Induce comparability failures (e.g., dual-task load, feature-binding interference, spatial integration disruption) while keeping local processing largely intact; assess whether conscious engagement degrades disproportionately relative to within-domain performance.

### 7.3 Prediction 3: cross-domain settlement

If conscious engagement reflects settlement control coordinating across heterogeneous domains, then intensity of conscious engagement should coincide with coordinated shifts in arbitration parameters across multiple subsystems, not diffuse or global activation. The key prediction is cross-domain coupling (covariation) of arbitration parameters, such that priority, urgency, confidence, and inhibition thresholds shift together across subsystems in a coordinated way.

At the network level, salience-related circuitry has been implicated in switching between control-related and default-mode networks during demanding task engagement, consistent with transitions into coordinated settlement modes rather than diffuse activation without functional agreement (Sridharan et al., 2008). Perturbational measures show that conscious activity covaries with integrated yet differentiated large-scale dynamics, not with activity that is locally bound or globally stereotypic (Casali et al., 2013; Mashour et al., 2020; Varley et al., 2020). Conversely, in some seizure states, generalized coupling and synchrony can increase while consciousness decreases, underscoring that “more coupling” is not the same as task-appropriate settlement (Guye et al., 2006; Blumenfeld, 2012).

Suggested test: Within individual subjects, simultaneously measure attention, autonomic state, motor readiness, and memory encoding while varying constraint; test whether conscious engagement covaries with task-specific coordination across these domains rather than undifferentiated synchrony.

### 7.4 Prediction 4: consciousness should degrade when cross-domain settlement is unnecessary, disrupted, or replaced by different priorities

If conscious engagement reflects cross-domain settlement coordination, then engagement should relax or collapse when cross-domain policy selection is not the dominant priority, or the coordination substrate for settlement is disrupted.

Sleep is compatible with the first case. During sleep, the system’s dominant objective shifts toward internal maintenance (synaptic homeostasis, consolidation, cleaning, repair), where continuous coordinated settlement would interfere with long-run stability (Friston, 2010; Dehaene and Changeux, 2011). General anesthesia is compatible with the second. General anesthetics can preserve many local processors while disrupting large-scale coordination such that the system cannot sustain coherent cross-domain settlement (Alkire et al., 2008; Redinbaugh et al., 2020; Mashour et al., 2020; Varley et al., 2020; Mashour, 2022). Moreover, intermediate levels of anesthesia and sleep have been shown to deliver graded levels of consciousness (Casali et al., 2013; Varley et al., 2020). Flow states provide a complementary case in which cognitive competence can persist as the need for cross-domain coordination declines within a well-practiced activity. Flow has been shown to coincide with high performance, reduced self-consciousness, and a shift from explicit toward implicit control (Csikszentmihalyi, 1990; Gold and Ciorciari, 2020).

Suggested test: Compare states where cross-domain settlement is needed (e.g., newly learned complex skills), unnecessary (e.g., overlearned habits or flow states), or disrupted (sleep, anesthesia) using matched sensory stimulation; test whether local processing can persist while settlement signatures (structured covariation; differentiated integration) degrade.

### 7.5 Prediction 5: comparative biology should show signs of unevenly distributed and graded settlement control

If conscious engagement depends on cross-domain settlement, then lineages with sophisticated local control and even global integration may nonetheless differ in their capacity for cross-domain convergence. The variable that should distinguish levels of conscious engagement across lineages is the presence of a shared currency that makes heterogeneous control signals rapidly comparable for unified arbitration.

Primates show clear evidence for flexible problem-solving across novel contexts, metacognitive behaviors, and complex social cognition that requires tracking others’ goals and beliefs (Beran et al., 2016). Insects also show impressive local control and multimodal integration, including sensory convergence in mushroom body circuits (Stopfer, 2014; Yagi et al., 2016), sophisticated foraging decisions (MaBouDi et al., 2023), and decentralized navigation (Cruse and Wehner, 2011). These capacities can support high within-domain performance, but they do not by themselves demonstrate a shared currency that enables cross-domain settlement. Whether insects can implement rudimentary cross-domain control remains unresolved, though some have argued that insects possess a capacity for basic subjective experience (Barron and Klein, 2016). By contrast, primates show physio-cognitive control couplings that are consistent with a candidate “common-currency” variable being used across domains and are assumed to have comparatively high levels of conscious experience (Boly et al., 2013). Markers include pupil-linked arousal covarying with conflict and predictive of subsequent behavioral adjustment (Ebitz and Platt, 2015; Joshi et al., 2016), and interoceptive state variables which have been linked through modeling to metacognitive confidence (Allen et al., 2022). Grading within the primate clade is supported by metacognitive monitoring, which scales from imprecise confidence judgments in capuchins (Smith et al., 2020) to more flexible self-monitoring in great apes (Beran et al., 2016), consistent with increasingly differentiated cross-domain control.

Cephalopods offer a particularly instructive comparative case. Octopuses demonstrate behavioral flexibility, and there is evidence suggestive of self-awareness and awareness of others (Schnell et al., 2023; Birch et al., 2025), yet their nervous systems are radically distributed rather than brain-centered (Olson et al., 2025) and as such, their architecture may not generate highly integrated information or large-scale global broadcasting in the vertebrate sense. Selective control accommodates these anomalies more naturally. The relevant question is not whether control is centralized or how much information is integrated or broadcast, but whether distributed controllers converge on a shared arbitration format under constraint. Whether cephalopod architecture supports such convergence, and at what grain, remains an open empirical question that selective control makes tractable.

Selective control also carries implications for engineered organisms (biobots, chimeras, organoids) whose architecture can be manipulated independent of evolutionary history (Kriegman et al., 2020). If settlement capacity depends on the presence of a functional self-model, real resource constraints, and convergent arbitration across heterogeneous domains, then the degree of conscious engagement in a synthetic being should be predictable from those structural features rather than from biological provenance. This offers a principled basis for evaluating novel beings whose status cannot be inferred from phylogeny alone (Levin, 2022).

Suggested test: Compare across and within lineages, matched on local control sophistication (e.g., foraging optimality, sensory discrimination) but differing in cross-domain comparability; test whether the presence of a shared comparability variable predicts cross-domain behavioral flexibility. In synthetic morphologies, independently perturb body plan, bioelectric coupling, metabolic constraint, and sensorimotor closure to test which combinations produce settlement signatures. To make this test concrete, consider a matched drive-conflict task in which foragers trade a food reward against a calibrated predation or thermal risk, holding within-domain discrimination difficulty constant. Measure decision latency on conflict relative to matched non-conflict trials, an arousal index (pupil diameter in primates, ventilation rate in cephalopods, aminergic proxies in insects), and whether an arbitration weight learned under one drive pairing transfers to a novel pairing. Arousal-coupled latency costs and cross-pairing transfer should track settlement capacity while within-domain foraging optimality should not. The prediction fails if a lineage shows full transfer and tight arousal–confidence coupling with no other settlement signature.

### 7.6 Prediction 6: artificial agents should develop consciousness only under real constraint

Artificial intelligence, synthetic morphology, and bioengineered organisms provide domains for experimental manipulation unavailable in evolutionary biology because constraints can be turned on and off (Dehaene et al., 2017; Butlin et al., 2025; Kriegman et al., 2020). If consciousness is a function of cross-domain settlement under constraint, then artificial systems should develop consciousness only when they must maintain the conditions of their own continued operation while arbitrating across competing policies under limited resources and irreversible risk of self-damage. The relevant pressure need not be evolutionary in origin. What matters is not how a system acquired its stakes but whether those stakes are owned by the system itself. Engineering does not preclude ownership: a designed system can have owned stakes if its continued operation actually depends on conditions its own regulation maintains, and if it also maintains the processes that perform that regulation. An objective merely assigned and scored from outside does not qualify, however real the budget it references, because the dependence must be physical rather than represented.

The core prediction is self-anchored cross-domain settlement under constraint. If the loss is only an external score or a resettable objective, no equivalent pressure is present. When an agent must maintain the conditions required for its own continued operation, errors can affect its viability. If the agent also faces real energy and computational constraints, models its boundaries, vulnerabilities, and internal state, and acts through closed sensorimotor loops, then purely local arbitration should increasingly fail under uncertainty, conflict, and irreversibility (Bongard et al., 2006; Ortega and Braun, 2013; Kriegman et al., 2020; Doncieux et al., 2015; Dehaene et al., 2017; Butlin et al., 2025; Seth, 2025). Prior work provides separate precedents for adaptive network coordination, binding across distributed processors, and resource-sensitive decision-making (Stibel et al., 2009; Stibel, 2013; Baars et al., 2013; Ortega and Braun, 2013). The present account goes further by predicting adaptively thresholded settlement episodes, measurable as coordinated shifts in control parameters across subsystems rather than generalized increases in information processing.

Suggested test: In embodied artificial agents where diverse controllers compete for the same resources, toggle real constraints and constitutive self-maintenance requirements, impose intrinsic viability costs distinct from external incentives, and test whether successful architectures develop episodic, system-wide coordination of arbitration variables. Such tests bear on whether the ownership and settlement conditions are met, not on whether the system is conscious, which no experiment can establish.

### 7.7 Disconfirming patterns

Across these predictions, the selective control framework is weakened if (i) conscious intensity scales with sensory richness instead of constraint, (ii) disruptions in comparability do not reliably impair cross-domain control, (iii) conscious episodes fail to show cross-domain coordination, (iv) consciousness persists despite disrupted settlement capacity, (v) lineages with differing levels of conscious engagement show no differences in cross-domain settlement signatures, or (vi) consciousness is achieved in AI or synthetic morphology without constraint-sensitive, system-wide settlement.

## 8 Discussion

### 8.1 Selective control under constraint

Selective control under constraint offers a functional principle capable of explaining a range of phenomena that have resisted unification across disciplines: from subjective experience to executive control, from self-awareness to attention, from neural integration to cultural scaffolding. The control ladder runs from boundary preservation to cross-domain settlement. Consciousness is that settlement in a self-anchored system.

These claims yield three evolutionary implications: consciousness should be unevenly distributed across lineages, graded in scope across species within a lineage, and adaptively scaled within settlement-capable organisms. Phylogenetically, this shifts the central question from “which species have the right neural parts” to “which lineages show evidence of cross-domain settlement under constraint.”

Rather than resolving the “hard problem” directly (Chalmers, 1996), the selective control framework changes what needs explaining. By grounding consciousness in the evolutionary logic of self-preservation, this framework outlines an incremental trajectory from chemistry to cognition. There is no need for a sudden appearance of a novel faculty; only slight, successive refinements in control architecture as systems scale. Chemical asymmetries support primitive self-discrimination (Maturana and Varela, 1980; Kauffman, 1993); compartmentalization supports self-protection (Szostak et al., 2001; Lane and Martin, 2012; Alberts et al., 2014; Szathmáry, 2023); excitable neural signaling enables fast, targeted arbitration across tissues and timescales (Striedter, 2004; Friston, 2010; Feinberg and Mallatt, 2017); affect and inferential models compress internal states into a common currency for control-relevant signals (Craig, 2002; Singer and Damasio, 2025; Laukkonen et al., 2025); social identity extends self-preservation beyond the body to kin and group protection (Matzinger, 1994; Boehm, 2012); symbolic culture externalizes regulatory load (Coolidge and Wynn, 2018; DeSilva et al., 2023; Stibel, 2025); and settlement control provides the cross-domain coordination that binds these components into a coherent whole.

Existing mechanistic theories describe how aspects of conscious processing are realized rather than why the architecture is favored (Friston, 2010; Mashour et al., 2020; Tononi et al., 2016; Dehaene et al., 2006; Laukkonen et al., 2025). Selective control supplies the adaptive logic that motivates complex regulatory architectures and sharpens what to look for: constraint-sensitive scaling, cross-domain coordination, and characteristic cost signatures when a coherent settlement is required.

Selective control under constraint also offers a principled roadmap for artificial consciousness. If nervous systems and brains are high-bandwidth substrates for scaling arbitration under constraint, then functional analogs of this architecture can emerge in artificial or bioengineered systems that face comparable demands. Consciousness should be possible, but only in agents that must maintain themselves and the conditions their operations depend on, operate under real resource budgets, use a functional self-model, and act in closed loops where consequences change future viability (Bongard et al., 2006; Ortega and Braun, 2013; Butlin et al., 2025).

In this sense, consciousness is not evolution’s anomaly but evolution’s solution. Selective control gives the evolutionary problem its computational structure, and consciousness its experiential form.

## Data Availability

The original contributions presented in the study are included in the article/supplementary material, further inquiries can be directed to the corresponding author.
